# Sublethal exposure of small few-layer graphene promotes metabolic alterations in human skin cells

**DOI:** 10.1038/s41598-020-75448-0

**Published:** 2020-10-27

**Authors:** Javier Frontiñan-Rubio, M. Victoria Gomez, Viviana Jehová González, Mario Durán-Prado, Ester Vázquez

**Affiliations:** 1 Instituto Regional de Investigación Científica Aplicada (IRICA), Universidad de Castilla-La Mancha, 13071 Ciudad Real, Spain; 2 Cell Biology Area, Department of Medical Sciences, Faculty of Medicine, Universidad de Castilla-La Mancha, 13071 Ciudad Real, Spain; 3 Faculty of Chemical Science and Technology, Universidad de Castilla-La Mancha, 13071 Ciudad Real, Spain

**Keywords:** Cell biology, Risk factors

## Abstract

Small few-layer graphene (sFLG), a novel small-sized graphene-related material (GRM), can be considered as an intermediate degradation product of graphene. GRMs have a promising present and future in the field of biomedicine. However, safety issues must be carefully addressed to facilitate their implementation. In the work described here, the effect of sub-lethal doses of sFLG on the biology of human HaCaT keratinocytes was examined. A one-week treatment of HaCaTs with sub-lethal doses of sFLG resulted in metabolome remodeling, dampening of the mitochondrial function and a shift in the redox state to pro-oxidant conditions. sFLG raises reactive oxygen species and calcium from 24 h to one week after the treatment and this involves the activation of NADPH oxidase 1. Likewise, sFLG seems to induce a shift from oxidative phosphorylation to glycolysis and promotes the use of glutamine as an alternative source of energy. When sub-toxic sFLG exposure was sustained for 30 days, an increase in cell proliferation and mitochondrial damage were observed. Further research is required to unveil the safety of GRMs and degradation-derived products before their use in the workplace and in practical applications.

## Introduction

Graphene-related materials (GRMs) have appeared as promising elements in different fields from composites to electronics applications^1^. In biomedicine, GRMs have been employed in imaging^2^, drug delivery^3,4^, diagnosis^5^ and many others areas^6–12^. Nonetheless, there is currently a lack of data concerning the effect of GRMs on human health, especially when these materials interact with highly sensitive barriers such as skin. The toxic effects of GRMs are determined by characteristics such as size, surface functionalization and dispersion method^13–19^. In consequence, it is critical to carry out biological studies in which these materials are carefully characterized with the aim of understanding the physicochemical characteristics that govern biological responses^10^.

Additionally, not only primary GRMs should be considered, but special attention should be paid to their degradation sub-products as there is already some evidence that these materials degrade in the environment. The results of several studies have proven that GRMs can be degraded by a photo-Fenton reaction^20^. The photooxidation of graphene oxide (GO) produces intermediate products with reduced sizes, low molecular weight and small amounts of oxygen functionality^21,22^. For example, AFM images of GO samples degraded in sunlight^21^ show that the degradation of this GRM gives rise to small flakes of around 40 nm and XPS results revealed a notable decrease in the oxygen content with respect to initial GO samples.

We previously demonstrated that few layer graphene (FLG) and GO are toxic to human HaCaT skin cells cultured in vitro^23^. High and moderate concentrations of each GRM damage cells mainly by physical interaction with the membranes, which results in cell necrosis^23^. Moreover, these actions are correlated with significant metabolic changes^23^. Herein we report the effect of small FLG (sFLG), a GRM similar to FLG but with graphene flakes smaller than 100 nm and a lateral size distribution with a major component of around 40 nm. This fact, along with the low oxygen content, means that this material can be considered as a sub-product of a GRM degradative process. sFLG is produced by a green preparation approach using glucose as the exfoliating agent^24^.

Our results indicate that sFLG clearly differs from FLG in its effects related to toxicity. This smaller material does not have any toxicity at concentrations where FLG does, with its effects not being translated into any observable phenotype assayed with toxicity. However, a more detailed analysis, including various cellular and metabolic approaches, revealed profound changes triggered by sub-toxic doses of sFLG, which induce mild early mitochondrial alterations that are sustained for up to seven days. These changes are initiated by alterations in the homeostasis of cytosolic Ca^2+^, H_2_O_2_ or NADPH-oxidase 1 (NOX1) enzyme levels, which denotes mitochondrial overload. This overload is evidenced by a profound change in the metabolome and bioenergetics pathways. With the assistance of nuclear magnetic resonance (NMR)-based metabolomics and SeaHorse XFp (Agilent) equipment, we dissected many alterations that are not evident with other techniques, such as an imbalance between oxidative phosphorylation and glycolysis, and the preferential usage of energy from other carbon sources such as glutamine. This use of glycolysis, which is known as the Warburg effect, has been observed in the initial steps of carcinogenesis^25,26^, although it could also be observed in non-transformed cells^27^. The mild mitochondrial damage, in conjunction with the remodeling of metabolism and sustained reactive oxygen species (ROS) production, could indicate the initiation of a tumorigenesis process^28^. In order to study this potential effect in depth, the long term effects were analyzed. It was found that a small metabolic disturbance is maintained and that this is coupled with an increase in cell proliferation.

Although genotoxicity assays are still required, the results of this work show the relevance of refining our roadmap for interpreting data for a better understanding of the interactions between different GRMs and human barriers, with particular attention paid to metabolism and mitochondria even when an evident phenotype is not noticeable. These studies are also important because some GRMs have already been proposed for use in contact with skin, for example in textiles^29^ or wearable electronics^30,31^. Special care must be taken to study the degradation and release of these materials during service life.

## Results

### Characterization of sFLG

**Figure 1:**
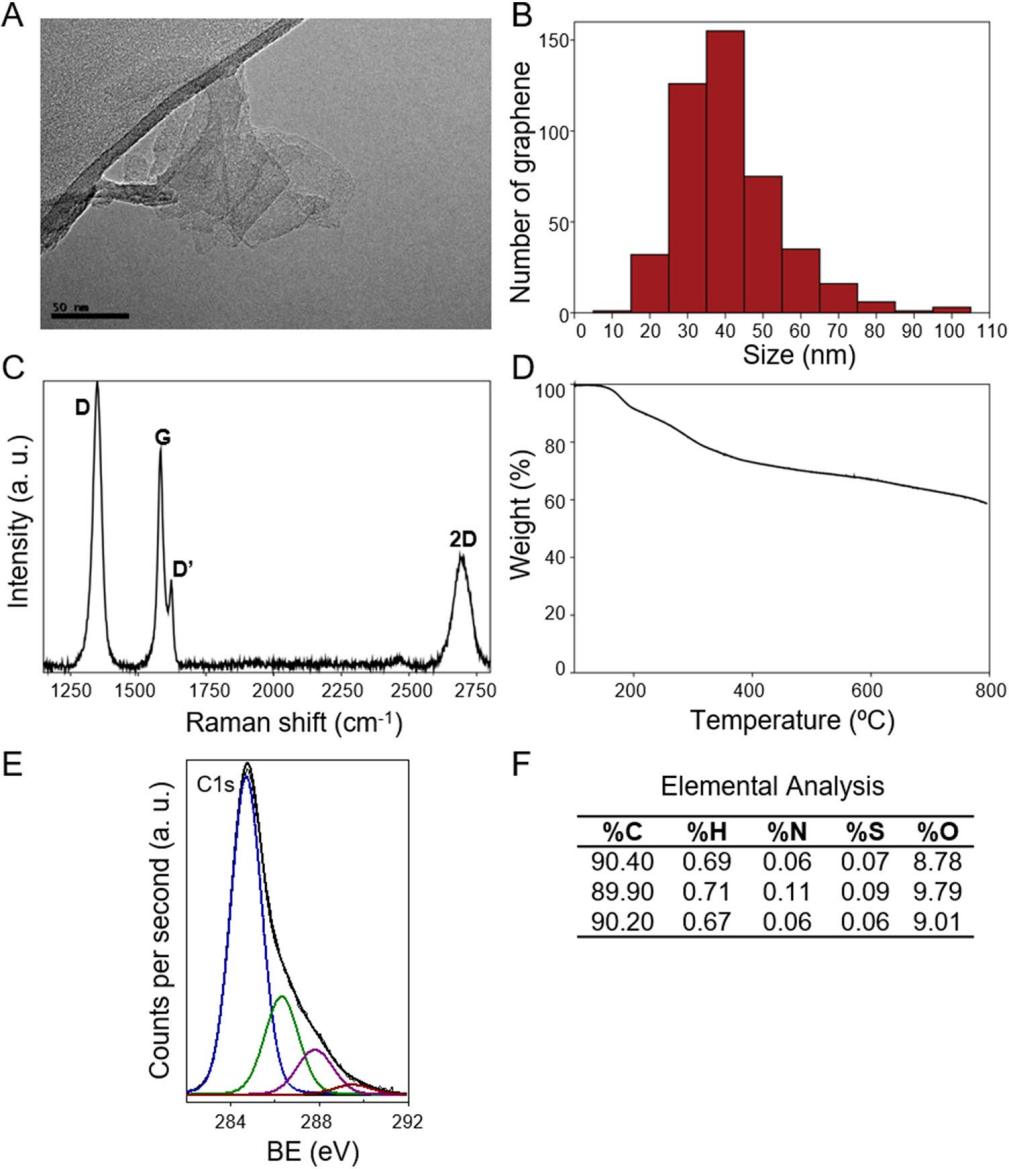
Characterization of sFLG by: (**A**) HRTEM Image, (**B**) size distribution of graphene sheets, (**C**) Raman spectra, (**D**) TGA results in a nitrogen atmosphere, (**E**) XPS results and (**F**) elemental analysis.

A typical image of sFLG is shown in Fig. 1A with graphene flakes smaller than 100 nm and a size distribution with a major component of around 40 nm (Fig. 1B). Raman spectroscopy was used to determine better the properties of these flakes. A representative Raman spectrum is shown in Fig. 1C, with characteristic graphene bands D, G, D′ and 2D observed (1350, 1580, 1600, and 2700 cm^–1^, respectively). The main band, the G band, exists for all sp^2^ carbon systems, the 2D band originates from the in-plane breathing-like mode of the carbon rings, and the D band is a disorder-induced band^32^. Meanwhile, D' appears as a shoulder on the G band and, like the D band, this also requires defects, although if the amount of disorder increases the G and D' bands can merge, as in the case of graphene oxide^33,34^. The relation between the intensities of the 2D (I_2D_) and G (I_G_) bands, and its full width at half maximum (FWHM), were used to determine the number of layers (NG) in sFLG^35–37^. The observed value I_2D_/I_G_ = 0.543 and the FWHM of 64 cm^–1^ are consistent with the presence of FLG^38^. Moreover, the information obtained from the 2D position and the equation described by Paton et al.^39^ allows an average number of three layers to be calculated. The intensity ratio between the D and G bands (I_D_/I_G_) (I_D_/I_G_ = 1.4) was used to quantify the density of defects in graphene^40,41^, but this value also correlates with the presence of small flakes. Meanwhile, XPS (Fig. 1E) was used to evaluate the functional groups in the sample. From the C1s spectra, it is possible to observe the presence of sp^2^ carbon bonds at 284.5 eV, C–O–C bonds at 286.4 eV, C=O bonds at 287.8 eV and C(O)O bonds at 289.3 eV, with a C/O ratio of 6.47. Thermogravimetric analysis (Fig. 1D) of sFLG performed under a nitrogen atmosphere is consistent with the XPS results, with a weight loss of 33% at 600 ºC due to the loss of residual oxygen-containing groups at the edges of the graphene sheets. Moreover, elemental analysis of sFLG gave average values of C (90.16 wt%), H (0.69 wt%), N (0.08 wt%), S (0.07 wt%) and O (9.00 wt%) (Fig. 1F). Finally, in order to determine possible metal traces, total reflection X-ray fluorescence (TXRF) was employed (Supplementary Fig. S1) and this confirmed the absence of metal contamination.

### Effects of sFLG on cell viability

**Figure 2:**
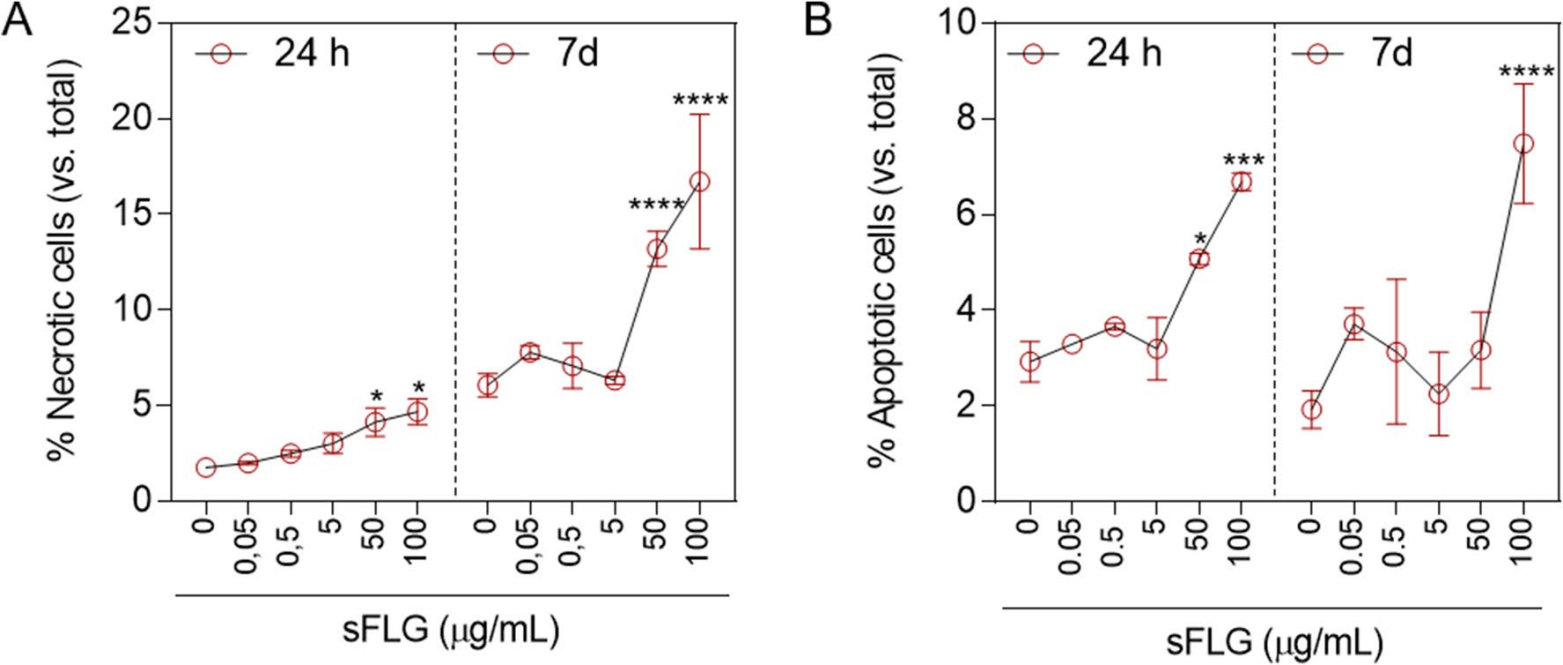
Effect of sFLG on HaCaTs necrosis and apoptosis. (**A**) Percentage of necrotic cells and (**B**) apoptotic HaCaT cells treated with sFLG for 24 h and 7 d (n = 3).

We previously reported that FLG and GO induce cytotoxicity in HaCaT cells in a time- and concentration-dependent manner, with necrosis induced and apoptosis initiated even at low concentrations (5 µg/mL) of each GRM^23^. Thus, as sFLG can be considered as a sub-product of FLG degradation, it was necessary to characterize its possible cytotoxic/cytostatic effects. The results presented herein show that incubation of human skin HaCaT cells with sFLG induces a dose- and time-dependent increase in necrosis and that this is higher at seven days incubation than at 24 h. However, this effect is only significant at high doses between 50 and 100 μg/mL, with values of 7% and 10% obtained, respectively (Fig. 2A). Similar results were obtained for apoptosis. A 24 h treatment with 50 and 100 μg/mL sFLG triggered apoptosis in 2% and 3.7% of cells, respectively, which increased slightly up to 5.5% with the highest dose after a seven day treatment (Fig. 2B). Therefore, 5 µg/mL was considered as a sub-toxic dose of sFLG since it does not increase apoptosis or necrosis. It was of great interest to evaluate the underlying cellular damage that sub-lethal doses can induce, since these will be the doses used in the different applications of GRMs.

### Effects of sFLG on metabolism

**Figure 3:**
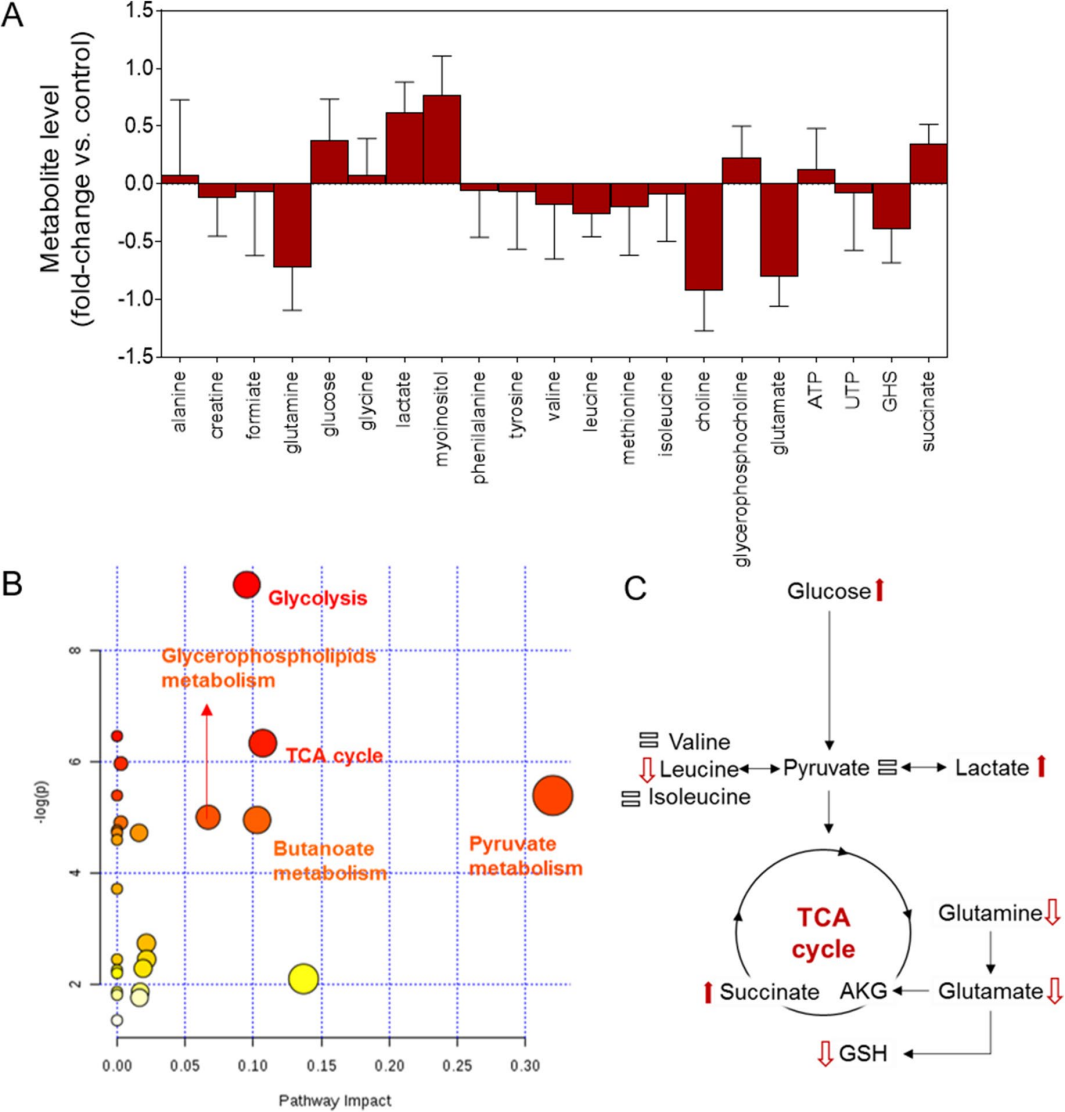
Impact of sFLG on HaCat cells metabolism. (**A**) Fold-change in metabolites induced by 7 d treatment with sFLG (n = 3). (**B**,**C**) main pathways altered.

Metabolomics is a powerful tool to evaluate the effects of nanomaterials on human cells^42–44^ as it allows the detection of metabolic changes that cannot be quantified by other techniques. An NMR-based metabolomics approach was previously used to demonstrate that low concentrations of GO and FLG (5 µg/mL) induce metabolic changes in HaCaT cells^23^. Following the same approach, we evaluated the effect of a low (5 µg/mL) sFLG concentration on human skin cells treated for up to seven days. Interestingly, although cell death was not observed under these conditions, metabolomics revealed a profound change in the metabolic profile of HaCaT cells. Specifically, increased levels of glucose, lactate, myoinositol and succinate by 0.37, 0.61, 0.76 and 0.34-fold *vs.* control were observed, respectively (Fig. 3A). Furthermore, glutamine, choline, glutamate and glutathione (GSH) levels were diminished by − 0.72, − 0.92, − 0.80 and − 0.39-fold *vs.* control, respectively (Fig. 3A). Enrichment analysis revealed an alteration in different metabolic pathways including those related to pyruvate metabolism, butanoate metabolism, glycerophospholipids metabolism, tricarboxylic acid (TCA) cycle and glycolysis (Fig. 3B,C). All of these pathways are critical for central metabolism and their alteration can affect directly or indirectly redox homeostasis, cell viability, motility and the whole cell bioenergetic status (Fig. 3B,C).

### Effect of sFLG on Ca^2+^ homeostasis

**Figure 4:**
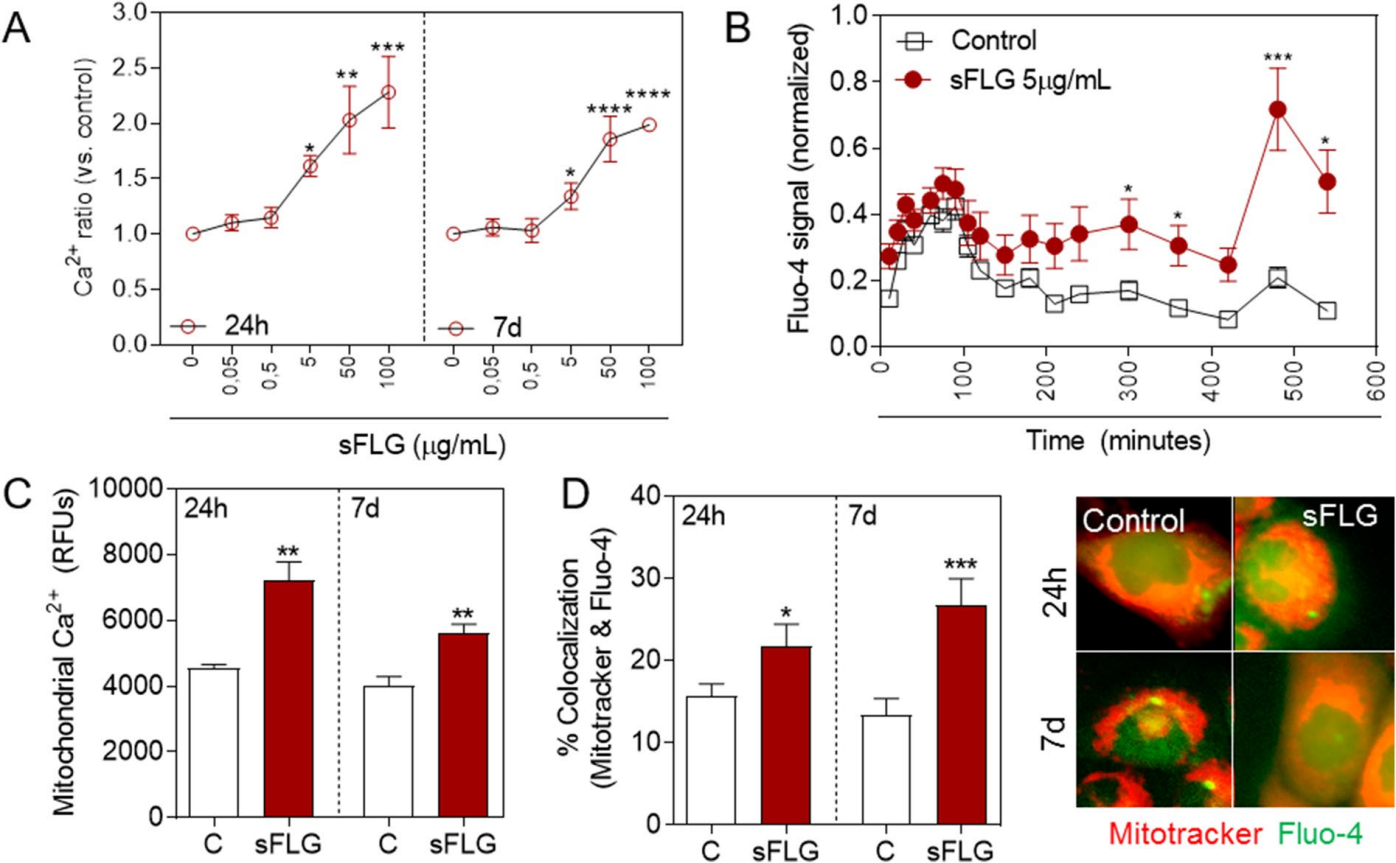
Effect of sFLG on free cytosolic and mitochondrial Ca^2+^ levels. (**A**) Fluo4-AM levels in cells treated with sFLG for 24 h and 7 d (n = 4); (**B**) Fluo-4 tracking in cells treated with sFLG 5 μg/mL (red) compared to control cells (black). (**C**) Mitochondrial calcium levels measured by quenching non-mitochondrial Calcein-AM (n = 3) and (**D**) % of colocalization between Fluo-4 and Mitotracker red in cells treated with 5 μg/mL sFLG for 24 h and 7 d (> 50 cells).

Ca^2+^ acts as a pleiotropic second messenger that triggers numerous and diverse physiological processes related to cell proliferation, vesicle transport, general homeostasis, oxidative stress and differentiation, amongst others^45^. In a previous work we observed that Ca^2+^ homeostasis in keratinocytes is disrupted upon treatment with GO and FLG, with the level of this second messenger increased after a 24 h treatment with 5 µg/mL of the GRMs, and this led to cell death^23^. The same approach was used to evaluate the effect of sFLG on Ca^2+^ levels in HaCaTs. sFLG raised the level of free cytosolic Ca^2+^ by more than 50% after treatment for 24 h, with 5 µg/mL being the lowest effective concentration (Fig. 4A). Higher sFLG doses increased the levels of free cytosolic Ca^2+^ to a greater degree and this was maintained up to seven days (Fig. 4A). In an effort to gain more insights into Ca^2+^ kinetics in response to sFLG, a real time experiment was carried out in which the variation in Ca^2+^ was monitored from the addition of 5 μg/mL of the GRM, i.e., the lowest effective concentration. This approach revealed that the disruption of Ca^2+^ homeostasis began very soon after sFLG addition, with a significant difference observed after only five hours of exposure (Fig. 4B).

The effect of sFLG on mitochondrial Ca^2+^ was monitored upon exposing HaCaTs to 5 μg/mL for 24 h and seven days. The samples were then stained with calcein-AM and CoCl_2_ to quench the fluorescence outside of the mitochondria by following a previously reported methodology^46^. This methodology indicated that mitochondrial Ca^2+^ levels were almost doubled 24 h after treatment and this increase was maintained after incubation for seven days (Fig. 4C). In order to confirm these data, the colocalization between Ca^2+^ levels (Fluo-4) and the Mitotracker-Red signal was analyzed. It was observed that the percentage of colocalization was doubled in cells exposed to sFLG for seven days (Fig. 4D), thus corroborating by indirect and direct methods the alteration of the homeostasis of mitochondrial calcium by sFLG sub-toxic exposure.

### ROS homeostasis alteration by sFLG

**Figure 5:**
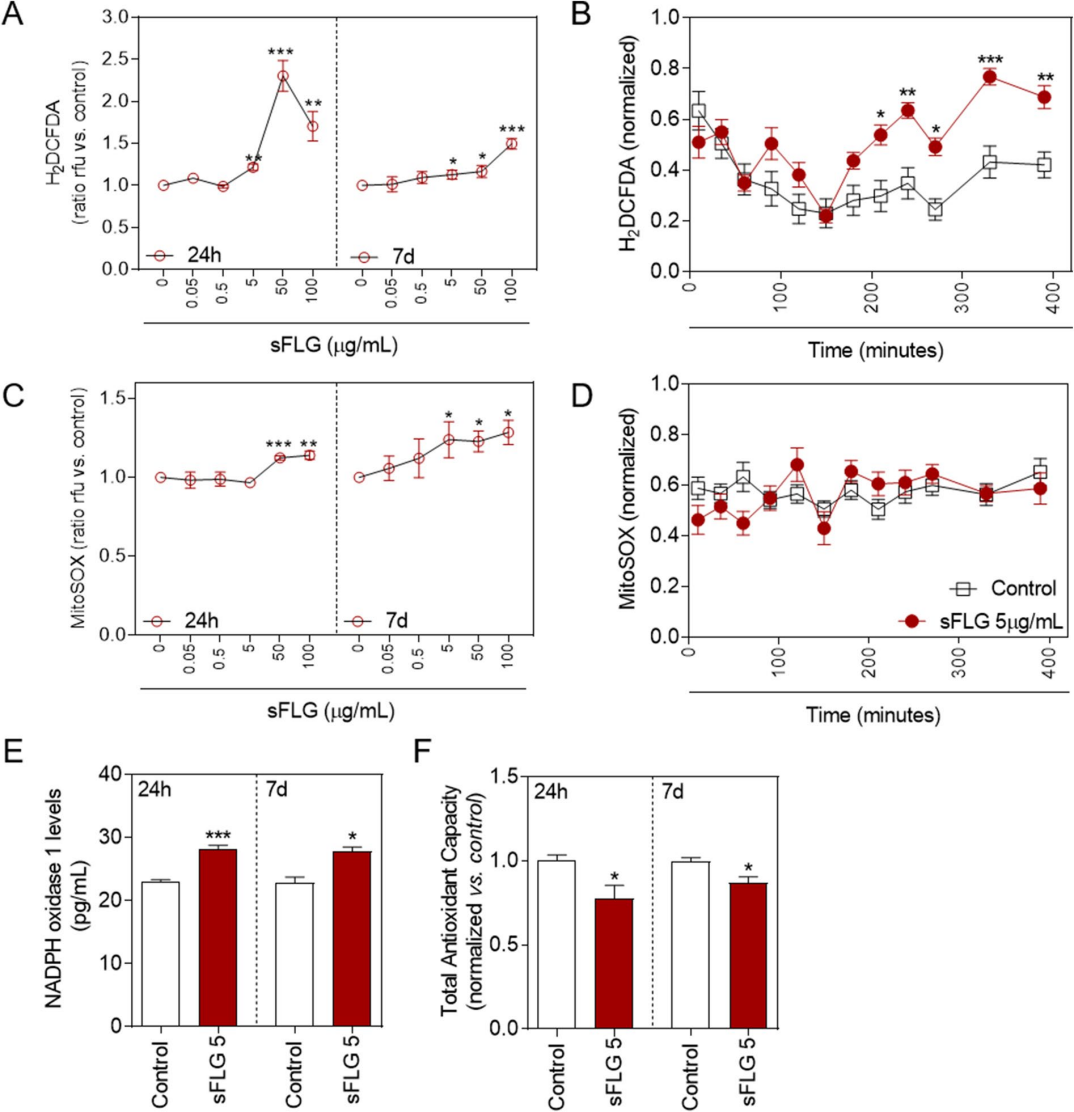
Impact of sFLG on H_2_O_2_ and O_2_^•−^ levels. (**A**) H_2_DCF-DA (H_2_O_2_) levels in cells treated with sFLG for 24 h and 7 d (n = 4). (**B**) H2DCF-DA tracking in cells treated with 5 μg/mL sFLG. (**C**) MitoSOX-AM (O_2_^•−^) levels in cells treated with sFLG for 24 h and 7 d (n = 4). (**D**) MitoSOX-AM tracking in cells treated with 5 μg/mL sFLG. (**E**) NADPH oxidase 1 levels (pg/mL of cell culture supernatant) and (**F**) total antioxidant capacity of cells treated with 5 μg/mL sFLG for 24 h and 7 d (n = 3).

ROS and Ca^2+^ are essential and interconnected signaling molecules in homeostatic/normal cell conditions^47^. However, increased levels of Ca^2+^ and overproduction of ROS are linked to diverse cellular processes, from metabolic alterations to cell death^48,49^. The effect of sFLG on ROS (H_2_O_2_ and mitochondrial O_2_^.–^) production was evaluated in HaCaT cells treated for 24 h and seven days with increasing concentrations of sFLG. The results indicate that sFLG raised the level of H_2_O_2_ at 24 h in a concentration-dependent manner, with a significant increase from 5 μg/mL (Fig. 5A, left) that was sustained to seven days (Fig. 5A, right). The results were similar for mitochondrial O_2_^.–^ (Fig. 5C), although the lowest concentration of sFLG (5 μg/mL) only increased O_2_^.–^ after seven days of incubation.

To gain further insights into the ROS kinetics in response to sFLG, a real time experiment was carried out to monitor the variation of H_2_O_2_ and mitochondrial O_2_^.–^ from the addition of 5 μg/mL of the GRM. The results show a significant increase in H_2_O_2_ at 210 min from the addition of sFLG, with this increase maintained up to the end of the experiment, i.e., eight hours (Fig. 5B). In contrast and supporting previous results at 24 h and seven days, a change in the mitochondrial O_2_^.–^ was not observed (Fig. 5D). This shows that sFLG had a biphasic effect by acting first on the membrane and cytosol, thus increasing the level of Ca^2+^ and H_2_O_2_, and secondly by exerting mild damage to mitochondria, which is translated into metabolic alteration at longer incubation times.

### sFLG effect on the oxidative balance

The results showed that the treatment of HaCaT with sFLG for 24 h downregulated the level of GSH and increased the level of H_2_O_2_, with the level of mitochondrial O_2_^.–^ remaining unaffected.

The above evidence allowed us to evaluate the possible modulation of NOX1 by sFLG, a key component of the redox homeostasis system^50^. To that end, HaCaTs were exposed to 5 μg/mL of the GRM for 24 h and seven days. The results showed a 20% increase in the level of NOX1 after a 24 h treatment and this was almost fully maintained up to seven days (Fig. 5E). This increase in NOX1 could be a protective cell mechanism to an external injury, as NOX activation has been described in the degradation of carbon nanotubes^51^.

All of the above data suggest that sFLG is altering the oxidative balance. In an effort to clarify this point, the effect of the GRM on the total antioxidant capacity (TAC) of the cells was evaluated. A significant reduction in TAC levels was observed after 24 h and this was maintained up to seven days (Fig. 5F).

### Impact of sFLG on mitochondrial respiration

**Figure 6:**
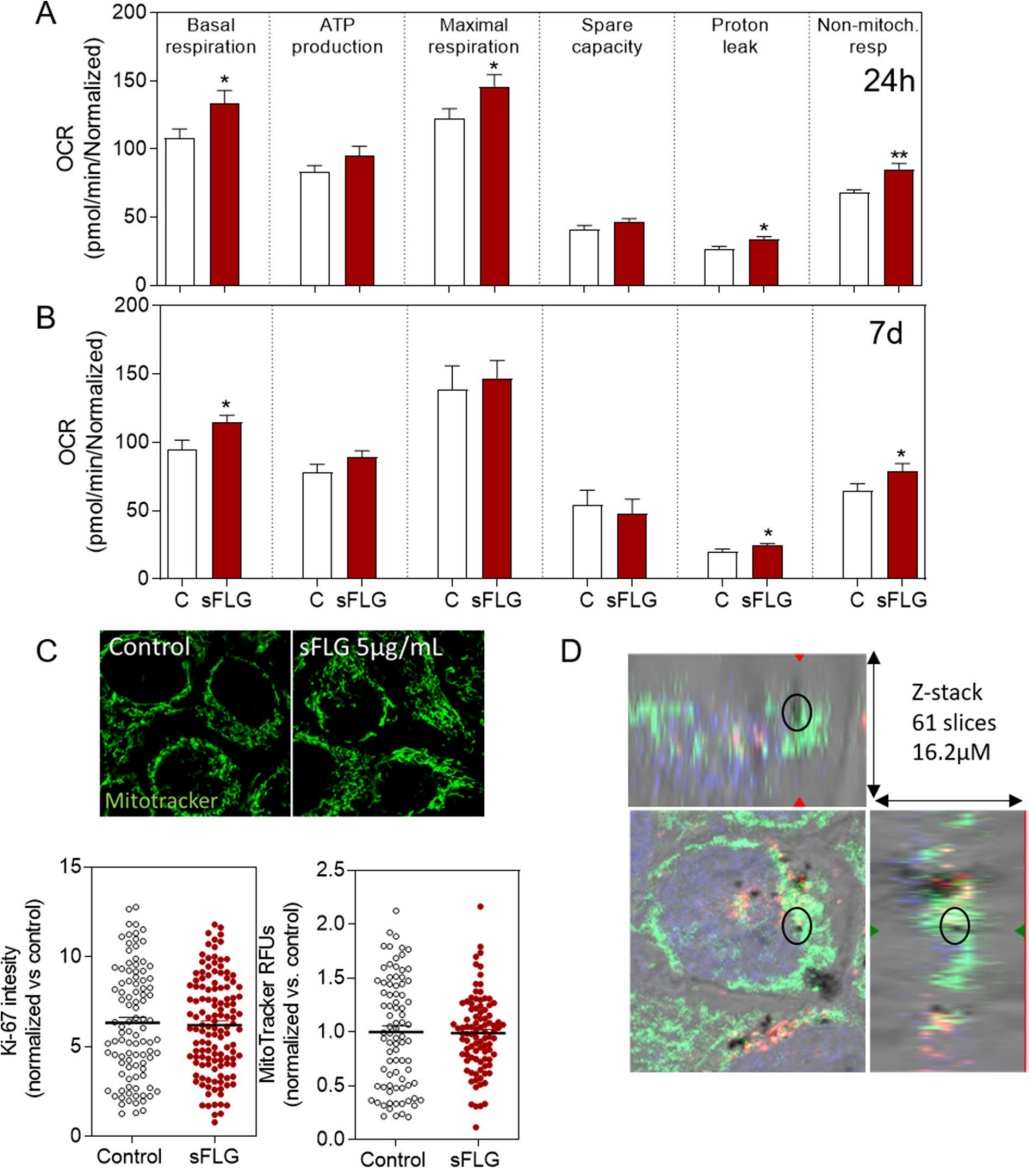
Effect of sFLG on mitochondrial respiration. (**A**) and (**B**) Individual parameters for basal respiration, ATP production, maximal respiration, spare respiratory capacity, proton leak and non-mitochondrial respiration in HaCaT cells treated with 5 μg/mL sFLG for 24 h or 7 d (n = 4). (**C**) Percentage of cell area occupied by mitochondria and intensity of Mitotracker staining in cells treated with 5 μg/mL sFLG for 7 d (nº cells > 100). (**D**) Z-stack (61 slices) of cells stained with MitoTracker Green treated with 5 μg/mL sFLG (black dots) for 24 h.

In order to gain further insights into the changes in the mitochondria and the overall effect on bioenergetics, oxidative respiration assays were performed on HaCaT cells, exposed to 5 μg/mL, using a Seahorse XFp extracellular flux analyzer and a Cell Mito Stress test kit (Agilent) (Fig. 6A,B) according to previously reported protocols^52^. As a first approach we evaluated the effect of sFLG at very short term, i.e., six hours, but did not observe a difference in any of the parameters analyzed (Supplementary Fig. 2). However, a 24 h treatment increased the oxygen consumption ratio (OCR) due to an increase in basal respiration and maximal respiration, which were accompanied by a parallel increase in proton leak without affecting ATP production (Fig. 6A).

Similar results were obtained after incubation for seven days (Fig. 6B). Although it was not probable, it was possible that sFLG would affect the total number of mitochondria. In an effort to clarify this possibility, HaCaT cells treated for seven days with 5 μg/mL sFLG were stained with MitoTracker green to analyze the mitochondrial cell area and the signal intensity of the probe. Neither of the parameters were affected (Fig. 6C) and this indicates that the number of mitochondria per cell remained unaffected. Confocal microscopy with a 63 × objective was employed to carry out a z-stack acquisition of whole cells treated for seven days with 5 μg/mL sFLG and control cells (Supplementary Fig. 3). Colocalization was found between graphene aggregates (black dots; Fig. 6D and Supplementary Fig. 2) and mitochondria (Mitotracker Green, Fig. 6D and Supplementary Fig. 3).

### Impact of sFLG on glycolysis

In an effort to evaluate the complete roadmap of alterations induced by sFLG on HaCaT cells, the glycolytic function was analyzed by measuring the extracellular acidification rate (ECAR) using a saturating concentration of glucose (10 mM) to increase ECAR followed by oligomycin treatment to inhibit ATP synthase.

**Figure 7:**
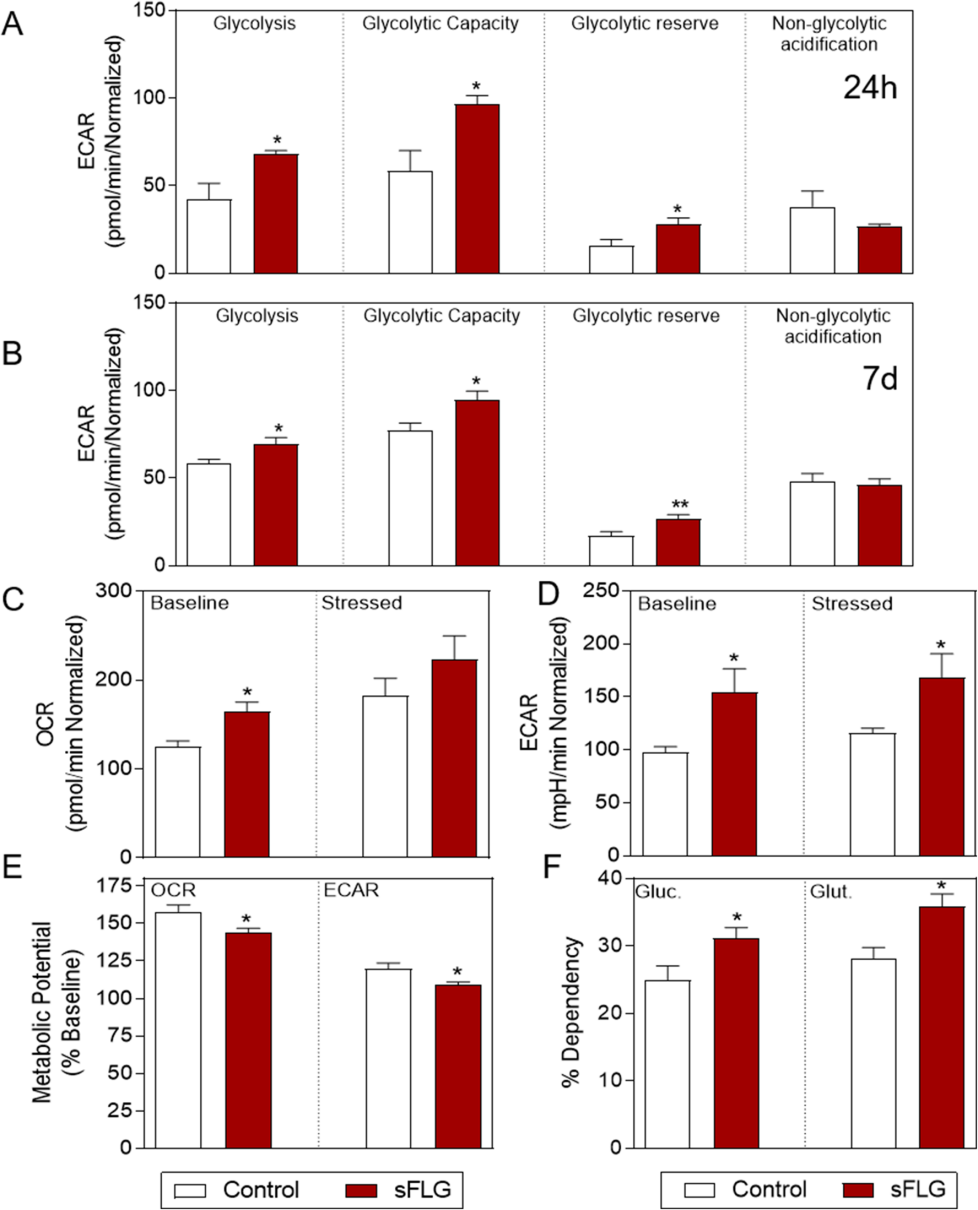
Glycolysis alterations induced by sFLG. (**A**) and (**B**) Individual parameters for glycolysis, glycolytic capacity, glycolytic reserve and non-glycolytic acidification in cells treated with 5 μg/mL sFLG for 24 h or 7 d (n = 4). (**C**) and (**D**) OCR and ECAR levels under baseline and stressed conditions, (**E**) metabolic potential and (**F**) percentage of glucose and glutamine dependency in cells treated with 5 μg/mL sFLG for 7 d (n = 4).

The results of these experiments revealed that a 24 h treatment of HaCaT cells with 5 μg/mL sFLG doubled glycolysis, glycolytic capacity and cell glycolytic reserve without affecting non-glycolytic acidification (Fig. 7A). These results were maintained up to seven days, the endpoint of the experiment (Fig. 7B), thus indicating the triggering of glycolytic machinery. However, the question remained as to whether the increase in OCR and ECAR rates would be maintained in an energy-demand condition.

The OCR and ECAR were then measured under baseline and stress conditions. In this scenario, OCR increased under baseline conditions upon sFLG treatment, but there was no significant alteration under stress conditions (Fig. 7C and Supplementary Fig. 4). In contrast, ECAR increased both under basal and stress conditions, thus indicating a predominance of glycolysis *vs.* oxidative phosphorylation (Fig. 7D and Supplementary Fig. 4). This shift was mirrored by a similar decrease in the cell metabolic potential, which determines the ability of cells to meet an energy demand through respiration and glycolysis (Fig. 7E). In addition, substrate-dependency assays revealed that this same treatment increased the cell dependency of glucose and, in particular, glutamine (Fig. 7F).

### Effect of sub-chronic sFLG incubation

**Figure 8:**
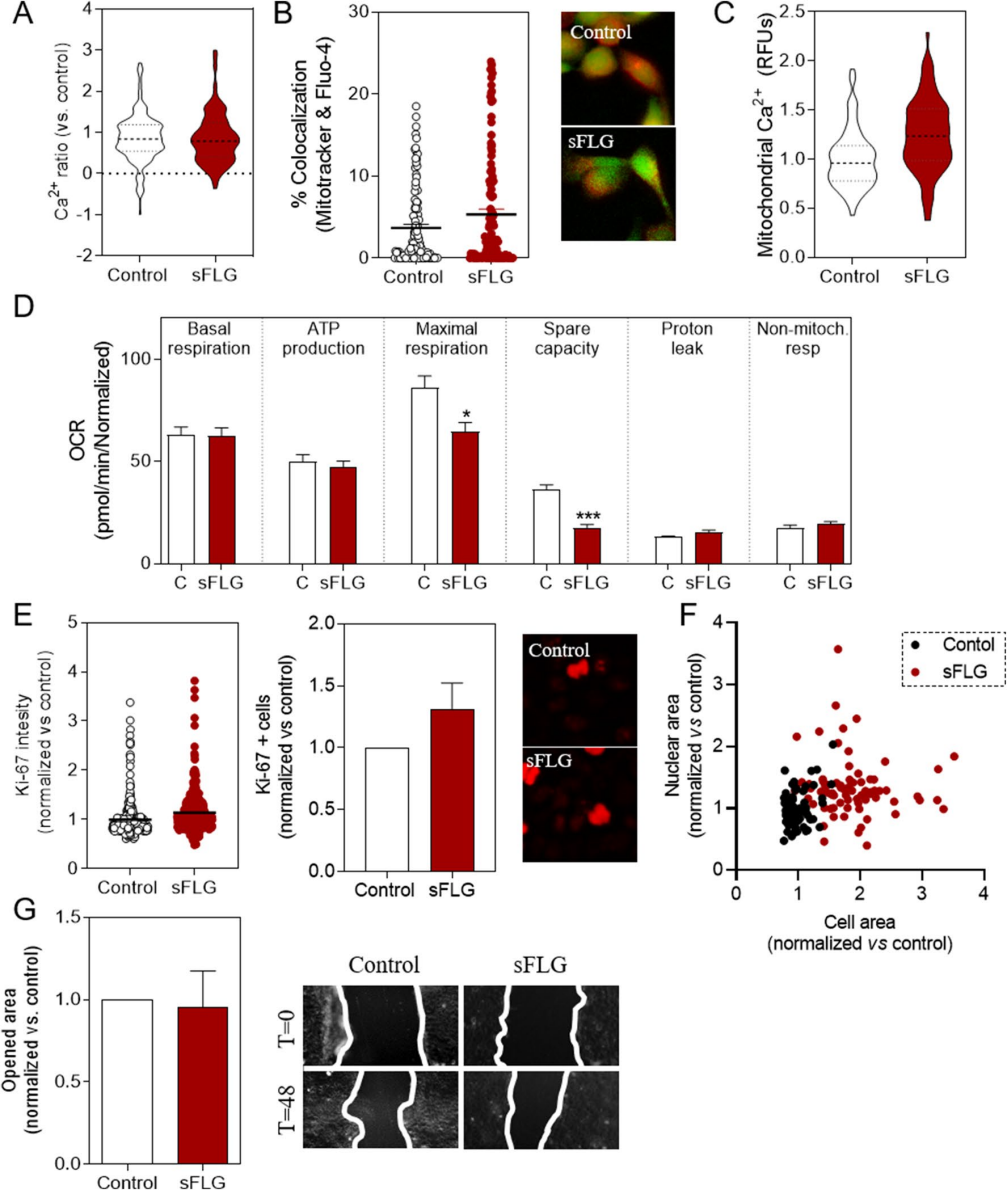
Long-term effects of sFLG. (**A**), Fluo4-AM levels in HaCaT cells treated with sFLG for 30 d (n = 3). (**B**) mitochondrial Ca^2+^ level evaluated by quenching cytosolic Calcein-AM and (**C**) colocalization between Fluo-4 and Mitotracker red levels. (**D**) Individual parameters for basal respiration, ATP production, maximal respiration, spare respiratory capacity, proton leak and non-mitochondrial respiration in cells treated with 5 μg/mL sFLG for 30 d (n = 3). (**E**) Ki-67 intensity and number of positive cells in control and in cells treated with 5 μg/mL sFLG for 30 d (> 50 cells). (**F**) Nuclear and cell area of control and treated cells with 5 μg/mL sFLG for 30 d (> 50 cells). (**G**) The percentage of open areas normalized in control and cells treated with 5 μg/mL sFLG for 30 d (n = 3).

As a first approach to ascertain the effect of long-term treatment with sFLG, a study was performed on the level of mitochondrial Ca^2+^, the energy status and some phenotypic characteristics in HaCaT cells treated for 30 days with 5 μg/mL sFLG. It was noticeable that there was no change in the free cytosolic Ca^2+^ level in cells exposed to 5 μg/mL for 30 days (Fig. 8A). However, mitochondrial Ca^2+^ had increased at this time by a similar magnitude to the change observed upon treatment for *seven days* (Fig. 8B,C). Therefore, the mitochondrial alteration was maintained upon long-term sFLG exposure. To gain more insight into mitochondrial changes, the mitochondrial stress was evaluated by Seahorse XFp technology. This approach revealed that maximal respiration and spare capacity decreased upon treatment with sFLG (Fig. 8D). There is, therefore, a change in the mitochondrial condition observed in cells treated for 24 h and seven days.

All of the alterations in the overall features of cellular and mitochondrial homeostasis discussed above could be translated into functional changes. In this sense, several parameters of particular interest were evaluated^53,54^. Ki-67 is a widely used and approved cellular marker for proliferation^55^. Ki-67 levels and the number of positive cells were evaluated, with a non-significant increase in both intensity level and number of positive cells observed (Fig. 8E).

The cell and nuclear size in sub-chronic exposed cells were subsequently analyzed and a marked difference in morphology was observed between control and sFLG-treated cells (Fig. 8F). sFLG induced a slight increase in nuclear size (Supplementary Fig. 5) but a much more pronounced increase in total cell size (Supplementary Fig. 5). As a first approach to functional changes, the migration was studied but differences between control and sFLG-treated cells were not observed (Fig. 8G).

## Discussion

sFLG can be considered as a degradation product of GRMs due to its size and physicochemical characteristics. Graphene is a promising candidate for the preparation of novel materials for multiple applications, which makes it essential to understand and dissect the mechanisms that govern its toxic effects and to determine the exact toxicity range for each GRM. The results presented above clearly indicate that sFLG is less toxic than FLG and GO^23^ and it can be considered ‘safe’ because in HaCaT cells it did not have a noticeable effect in terms of an increase in cell death detected by classical toxicological/microscopy techniques. Besides, these results confirm that the main cytotoxic damage of FLG and GO, at least to epithelial cells, is due to physical disruption of the plasma membrane, as published in earlier works^23,56,57^. However, special attention should be paid to the effects that sFLG has on metabolism. The results indicate that treatment of HaCaT cells with sub-toxic concentrations of sFLG induces mild mitochondrial damage preceded by a cytosolic Ca^2+^ overload and a parallel increase in oxidative stress.

There is evidence to indicate that GO alters proteins related to energy metabolism in breast cancer cells, specifically those related to oxidative phosphorylation and the TCA cycle^58^. Moreover, we previously reported that these pathways are altered by GO and FLG in HaCaT cells and that this is linked to a pro-death phenotype^23^. GO and FLG can damage mitochondria and induce HaCaT cell death upon acute treatment^57^ and it is possible that sFLG also alters the organelle and exerts moderate damage that is not sufficient to trigger either apoptosis or necrosis.

This mild mitochondrial damage caused by sFLG is also supported by the results on mitochondrial Ca^2+^ levels, the deregulation of which could affect the whole cell bioenergetics^59^. This factor should be considered for future uses of GRMs, as one of the envisaged biomedical applications of these compounds is to deliver drugs into mitochondria^60,61^.

Another indirect clue for mitochondrial damage is the activation of the NOX1 enzyme in response to sFLG, as described herein. The activation of this enzyme could be the key to revealing why the level of H_2_O_2_ increased in the early stages, where mitochondria are not yet severely damaged. Indeed, Sun et al. reported previously that carbon nanotubes can induce NOX activity in vitro^62^. Recently, Pelin et al. proposed a putative mechanism for mitochondrial damage induced by FLG and GO in HaCaT skin keratinocytes, whereby the GRM-induced ROS increase is mediated by the activation of flavoprotein-based oxidative enzymes^57^. In the same work, the authors observed indirectly (using a NOX inhibitor) that NOX was not altered. In our case, an alteration was observed in the activity of this enzyme and this could indicate a differential effect of the sFLG.

sFLG not only increases H_2_O_2_ levels but also affects the whole redox balance by decreasing the level of antioxidant molecules such as GSH. It has previously been reported that GO decreases the level of GSH in zebrafish and rat kidneys^63,64^. A decrease in GSH is also linked to FLG-induced cytotoxicity in HUVEC cells^65^. GSH prevents oxidative stress and a drop in the levels of this compound could be fatal if cellular ROS levels are increased due to external agents^66^. Thus, sFLG could be disarming antioxidant HaCaT components as a decrease in the level of GSH and its precursors was observed (Fig. 4).

sFLG also cuts glutamine levels and induces a double impact in cell homeostasis. Firstly, a reduction in glutamine levels could also be indicative of an enhanced consumption of the amino acid – a consequence of an adaptative response to mitochondrial stress^67^. Secondly, glutamine and glutamate, which is also diminished, are key metabolites for the synthesis of GSH^66^.

The results reported here for sFLG indicate metabolic changes that precede more evident cellular alterations. In this scenario, the observed enhanced levels of glucose and lactate are striking and suggest a shift from oxidative phosphorylation to aerobic glycolysis, a change that is called the Warburg effect – a metabolic alteration observed in tumor cells^68,69^. OCR is also increased and this also supports mitochondrial damage and the start-up of bioenergetics mechanisms that are not related to oxidative phosphorylation. Overall, these results confirm that mitochondrial biology is being affected, although this process must be studied carefully as the observed metabolic change shares more characteristics with tumor metabolism than one might expect^25^. Specifically, the metabolic remodeling and cellular events altered by sFLG resemble the changes produced by UV in skin cells in the early stages of tumorigenesis before any evident cell transformation occurs^26^. It has recently been reported that these skin pre-tumor cells, even though phenotypical alterations have not yet occurred, show a metabolic remodeling that precedes transformation into tumor cells, which is related to an increase in lactate and glycolysis and an enhancement in glutamine dependency^26^. These facts do not necessarily mean that the same situation is arising in our case, but the indications highlighted in this work mean that this aspect must be studied more carefully. The reliance on glucose and glutamine of HaCaTs treated with sFLG by was assessed by quantifying mitochondrial respiration to determine how dependent the cells are on the pathway of interest to meet basal energy demand. The results show increased dependency on glucose and glutamine in response to sFLG (Fig. 8F), thus indicating that mitochondria require higher amounts of glucose to maintain basal respiration, due to the glycolytic shift, and that cells use other energy sources such as glutamine to compensate for this high energy demand. These metabolic changes occur very early, i.e., after a 24 h treatment, and last for seven days, which indicates that cells are not able to overcome the injury. If these metabolic changes are maintained over prolonged periods of time, they may dramatically affect cell homeostasis and lead to tumor features^70^. This is why one of our main goals in the laboratory today is to draw conclusions about the effect of sub-chronic exposure to GRMs.

As a first approach, we observed how the effect of sFLG on HaCaT cells is time-dependent. Interesting alterations have been identified in a sub-chronic exposure of 30 days to sFLG. Therefore, the time of exposure is also a key factor in GRM-induced toxicity. The decrease in maximal respiration (Fig. 8D) may be due to accumulated damage related to the increase in mitochondrial calcium overload for 30 days (Fig. 8B,C). Changes in proton leak in the longer term could be produced by alterations in the surface area and proton permeability of the mitochondrial inner membrane^71^, which is also directly related to Ca^2+^ homeostasis^72^. The changes observed in mitochondrial respiration may be related to the Warburg effect, which is responsible for an increase in cell proliferation^73^. The results reported here showed an increase in Ki-67 positive cells that, while not significant, indicated a trend towards increased cell proliferation (Fig. 8E). In addition to these results morphological changes were observed (Fig. 8F). Overall, small clues were observed that could be indicative of some tumor capacity of sFLG and we are currently carrying out a complete approach to study this potential, not only with sFLG but with other GRMs with different degrees of oxidation and lateral sizes.

A range of cellular changes generated by sub-lethal doses of sFLG have been characterized. The effects reported in this work have not been observed previously. This suggests that the graphene-cell relationship is much more complex than expected. Although more studies must be performed, this shift to a sustained pro-oxidant state in which GSH is decreased and O_2_^.–^ is augmented leads one to speculate that the sFLG could be inducing in cells a similar effect to that exerted by UV^26^ or even radiotherapy. This could lead to DNA damage and probably to the accumulation of DNA mutations^74^. Preliminary evidence already exists for DNA damage generated by different GRMs^75–77^ and it is worth highlighting the relevance of lateral size, oxidation, dose, etc. in DNA damage. It is therefore essential to assess whether these alterations can lead to tumor processes, especially with sub-lethal doses and sub-chronic exposures.

## Conclusions

The use of GRMs for future applications is promising but it is essential to study in depth the effects of the primary compounds as well as their degradation products. In the work described here we offer a more complete roadmap concerning the effect of sFLG on human skin cells. A powerful methodology is described to detect changes that are not evident with other classical techniques. It was observed that sFLG generates a whole series of changes in human skin cells, even on applying sub-lethal doses. In a multi-experimental approach, a remodeling of the cellular energetic metabolism was observed along with alterations in the calcium and redox homeostasis. This metabolic reshaping shares some characteristics with classic tumor cell metabolism. These alterations are also maintained when a sub-chronic exposure is carried out. Further assays at sub-chronic exposure are being conducted with different GRMs, with the aim of ascertaining the impact of prolonged elevated ROS levels and metabolic shift, as DNA stability could be compromised. The aim is to determine accurately the order of magnitude of the generated damage.

The effect of acute doses of GRMs at short exposure times is now well characterized both in vivo and in vitro^18,19^. The results of the present work show the relevance of sub-toxic doses at short times and sub-chronic exposures. Similar exposure levels can be found in the workplace or can be envisaged in many of the present and future applications of graphene. It is therefore essential and necessary to study these processes in depth in order to guarantee safe use of this extraordinary nanomaterial.

## Methods

### sFLG preparation and characterization

sFLG was prepared using a previously described protocol^24^. 75 mg of graphite (Bay Carbon, Inc.) and 4.5 g of glucose (Panreac) were mixed in a 250 mL stainless-steel grinding bowl (15 stainless steel balls, each with a diameter of 2 cm) at 250 rpm and 4 h using a Retsch PM 100 planetary mill in an air atmosphere. The resulting solid was dispersed in 100 mL of water, sonicated for 1 min and centrifuged at 1500 rpm for 15 min to remove graphite and subsequently dialyzed to remove the residual glucose. The final graphene dispersions were left to stand for 5 d at room temperature in an air atmosphere. The supernatant was collected and lyophilized at − 80 °C at a pressure of 0.005 bar to obtain sFLG.

In order to avoid possible metal contamination from stainless steel balls, 1 mg of sFLG was treated with 1 mL of HCl (37%). The mixture was sonicated for 30 s and centrifuged for 5 min at 4000 rpm. The supernatant was removed and the solid was washed again with HCl (37%), NaOH (6 M) and finally three times with deionized water. Dry powder samples were obtained after lyophilization at − 80 °C at a pressure of 0.005 bar.

Thermogravimetric analysis (TGA) was carried out using a TGA Q50 (TA Instruments) at 10 °C min^–1^ under a nitrogen flow from 100 to 800 °C.

Raman spectra were recorded on an InVia Renishaw microspectrometer (Renishaw plc, United Kingdom) equipped with a 532 nm point-based laser, with a 50 × objective and an incident power density below 1 mW µm^–2^ to avoid laser heating effects. Raman samples were measured in solid state under ambient conditions with at least 30–40 random points taken on the sample.

Elemental analysis was performed with a LECO CHNS-932 analyzer, with complete combustion of the sample with four doses of oxygen and quantification of the released gases by thermal conductivity.

The morphology and lateral size were analyzed with a High-Resolution Transmission Electron Microscope (HRTEM) JEOL 2100 at an accelerating voltage of 100 kV. Samples were prepared as dilute dispersions of graphene for dip-casting on Lacey copper grids (3.00 mm, 200 mesh), coated with carbon film, and dried under vacuum. Finally, the lateral dimension distribution was calculated using ImageJ 1.53 software (https://imagej.nih.gov/ij/) and probing at least 100 flakes in each case.

In addition, total reflection X-ray fluorescence of sFLG was performed using a Bruker-S2 PicoFox TXRF spectrometer.

### Cell culture

HaCaT cells, a human epidermal keratinocyte line, were maintained in DMEM culture medium (#D5796; Sigma-Aldrich) supplemented with 10% fetal bovine serum (FBS) (#F4135; Sigma-Aldrich) and 1% antibiotic/antimycotic (#A5955; Sigma-Aldrich) at 37 ºC in a 5% CO_2_ atmosphere. Cells were used up to the 15th passage.

### Cells exposure to sFLG

Cells were exposed to sFLG for 6 h, 24 h, 7 d or 30 d depending on the assay. For 7 d and 30 d incubation, cell cultures were maintained according to standard procedures. Cells received fresh medium every 3–4 days and were subcultured and treated with sFLG every 7 d (see Supplementary Fig. 6).

### Determination of apoptosis, necrosis and viability

Viability, necrosis and apoptosis were determined as reported previously^23^. Briefly, HaCaT cells were seeded in 96 well plates and incubated for 24 h and 7 d with sFLG ranging from 0.5 to 100 µg/mL. Cells were incubated with 10 μg/mL ethidium bromide (EtBr) (#46,067; Sigma-Aldrich) and 1 μM Calcein-AM (#C34852; Thermo Fisher). Viable (Calcein positive; green) and necrotic (EtBr positive; red) cells were determined by fluorescence microscopy using a Cytation 5 system (20 × objective; BioTek) and image analysis with ImageJ 1.53. Immediately after image acquisition, cells were fixed and permeabilized for 2 min in ice-cold methanol and stained with 1 μg/mL Hoescht 33,258 (#861,405; Sigma-Aldrich). Apoptotic nuclei were determined according to morphological criteria^23^. The results are presented as percentage of viable, necrotic or apoptotic cells *vs*. total (n = 3).

### Sample preparation and measurements for NMR experiments

NMR experiments (n = 3) were conducted as described before^23^. Briefly, cells were grown in 3 × 175 cm^2^ flasks and treated up to 7 d with 5 μg/mL sFLG. Cells were detached, rinsed twice in PBS and homogenized by sonication in D_2_O (#151,882; Sigma-Aldrich). NMR spectra were acquired using a Varian Inova 500 spectrometer operating at 499.77 MHz for ^1^H and at 125.678 MHz for ^13^C with a four-nucleus 5 mm 1H {^15^N-^31^P) PFG high-field indirect detection probe. Standard 1D spectra with water suppression (presat) were recorded as previously reported by us^46^ with a 8 k spectral width, 32 k data points, a 90º pulse width of 12 µs, a 4 s relaxation delay and 860 scans, at 298 K and using pulse sequences from the Varian library. Manual phase and baseline correction were applied to all 1D spectra for processing. 2D homo- and heteronuclear correlation experiments *J*-resolved, ^1^H-^1^H-TOCSY, ^1^H-^13^C HSQC were carried out at 298 K for NMR peak assignment on an 800 MHz (^1^H) Advance NMR spectrometer (Bruker, Billerica, MA, USA) equipped with a ^1^H, ^13^C, ^15^N cryoprobe and Z-gradients in conjunction with the Bruker library. In addition, matching 1D and 2D data to reference spectra in both the Human Metabolome Database (HMDB) and the Birmingham Metabolite Library (BML-NMR), and the use of Chenomx NMR Suite 8.2 were used to assist in peak identification. Addition of standard samples to the mixture of metabolites was carried out to confirm peak identification for selected metabolites. The NMR peak quantification was assessed by Simple Mixtures Analysis (SMA, Mnova 11.0) using a known concentration of TSP (0.1 mM) as standard, and by manual peak picking using Global Spectral Deconvolution (GSD) from Mnova. For a more accurate quantification of the metabolites, a separate sample tube containing only TSP at pH 7.4 was prepared and its ^1^H NMR spectrum was registered at 298 K and with similar acquisition parameters as in the sample study.

### Determination and tracking of O_2_^.−^, H_2_O_2_ and Ca^2+^ in single cells

Mitochondrial O_2_^.−^, total H_2_O_2_ and free cytosolic Ca^2+^ levels were determined as reported before^23^, using MitoSOX fluorescent probes (#M36008; Thermo Fisher), H2DCF-DA (#C6827; Thermo Fisher) and Fluo-4 (#F23917; Thermo Fisher). Briefly, HaCaT cells were seeded in 96 well plates and incubated up to 24 h or 7 d with sFLG in the range 0.5 to 100 µg/mL. After treatment, cells were washed twice with PBS then loaded for 30 min with the fluorescent probe (one independent probe per assay; 1 µM MitoSOX and Fluo-4; 2.5 µM H_2_DCFDA) and imaged with a Nikon TiU microscope (20 × objective). Pictures were analyzed and processed with ImageJ 1.53. The results show the percentage of cell signal *vs*. control (n = 4). Tracking experiments were performed by monitoring fluorescence levels at different times in the same cells using a Zeiss LSM-600 confocal microscope (63 × objective). Pictures were processed with Zen 2012 blue edition and ImageJ 1.53. Results show the variation of fluorescence from time 0 (> 25 cells/experiment).

### Mitochondrial Ca^2+^ measurement

Levels of mitochondrial calcium were quantified by two different approaches. Cells were seeded in 96 optical well plates (Eppendorf) and incubated for 24 h or 7 d with sFLG 5 µg/mL. In the first approach, cells were loaded for 30 min with 1 µM Calcein-AM (#C1430; Thermo Fisher) and 1 mM CoCl_2_ to quench the fluorescence corresponding to cytosolic Ca^2+^ as described before^46^. After washing in fresh medium, images were acquired in a Cytation 5 Reader (Biotek) using a 20 × objective. The results were calculated with ImageJ 1.53 and are expressed as relative fluorescence units (RFUs) for each treatment (n = 3). In the second approach, cells were loaded for 30 min with 1 µM Fluo-4 and Mitotracker Red CMXRos (#M7512; Thermo Fisher). After washing in fresh medium images were acquired in a Cytation 5 Reader (Biotek) using a 20 × objective. The percentage of mitochondrial area colocalized with Fluo-4 signal was calculated with ImageJ 1.53 (colocalization plug-in). Results are expressed as the percentage of mitochondrial area colocalized per cell for each treatment (> 50 cells).

### NADPH oxidase 1 quantification by Enzyme-Linked Immunosorbent Assay (ELISA)

HaCaT cells were seeded in 24 well plates and incubated for 24 h and 7 d with sFLG 5 μg/mL. The levels of NOX1 were determined from culture media using a commercial ELISA kit (#MBS167429; MyBiosource) following the manufacturer’s instructions. Briefly, cell supernatant of sFLG incubated cells were prepared and added to cells with 10 μL anti-NOX1 antibody and 50 μL streptavidin-HRP. The plates were covered with a seal and incubated for 60 min at 37 °C. The wells were washed and treated with reaction buffers for 10 min at 37 ºC. Finally, the optical density (OD) of each well was measured at 450 nm. Results are expressed as pg/mL cell culture supernatants compared with the standard provided (n = 4).

### Determination of total antioxidant capacity

HaCaT cells were seeded in 96 well plates and incubated for 24 h or 7 d with sFLG 5 μg/mL. Total antioxidant capacity was determined in culture media using a commercial kit according to the manufacturer’s instructions (#MAK187, Sigma-Aldrich) as described before^74^. Briefly, supernatants samples were added to wells, then Cu^2+^ Working Solution was added. The samples were mixed using a horizontal shaker and incubated for 90 min at room temperature in the absence of light. Absorbance was measured at 570 nm and compared with Trolox equivalents (ranging from 4–20 nmol/well). Trolox is a water-soluble vitamin E analog and serves as an antioxidant standard. Results are expressed as the nmol ratio *vs.* control (n = 4).

### Quantification of mitochondrial respiration

A Seahorse XFp Analyzer (Seahorse Biosciences, North Billerica, MA) was used to quantify OCR and ECAR, following the protocol set up previously by Divakaruni et al*.*^52^. HaCat cells were incubated for 24 h and 7 d with sFLG 5 μg/mL in DMEM and then in Seahorse XFp base medium without phenol red (#103,193–100 Seahorse Biosciences) with a density of 3 × 10^5 ^cells/well in XFp miniplates (#103,025, Seahorse Biosciences). The samples were incubated for 60 min at 37 °C without CO_2_ prior to loading into the Seahorse analyzer. Three baseline OCR values were obtained during the first 20 min and then the different mitochondrial inhibitors were added (oligomycin, 1 μM; carbonyl cyanide-*p*-trifluoromethoxyphenylhydrazone (FCCP), 0.3 μM; antimycin A and rotenone, 1 μM). After different injections, three OCR values were automatically measured by the Seahorse XFp software Wave 2.6 (https://www.agilent.com/en/products/cell-analysis/cell-analysis-software/data-analysis/wave-desktop-2-6). For normalization, cells were fixed and permeabilized for 2 min in ice-cold methanol and then stained with 1 μg/mL Hoescht. The number of cells per well was obtained using a Nikon Ti U epifluorescence microscope with a 2 × objective and counting with ImageJ 1.53. Data are presented as mean ± SEM for each time point in pmol per minute normalized to the number of cells per well (n = 5).

### Determination of cell energy phenotype

HaCat cells were incubated for 24 h and 7 d with sFLG 5 μg/mL in DMEM and then incubated in XFp medium following the same protocol as indicated above. For cell energy phenotype determination, three baseline OCR and ECAR measurements were taken for each well within the first 20 min and then oligomycin (1 μM) and FCCP (0.3 μM) were injected. Six OCR and ECAR values were automatically calculated. Data were calculated by the Seahorse XFp software Wave 2.6. Data are presented as mean ± SEM for each time point in pmol per minute normalized to the number of cells per well (n = 4) as described before^52^.

### Determination of glycolytic stress

HaCat cells were incubated for 7 d with sFLG 5 μg/mL in DMEM and were then incubated in XFp medium, without glucose, following the same protocol as indicated above. Three baseline ECAR measurements were taken for each well within the first 20 min and glucose (10 mM), oligomycin (1 μM) and 2-deoxyglucose (50 μM) were subsequently injected. Three ECAR values were automatically calculated after each injection by the Seahorse XFp software Wave 2.6. Data are presented as mean ± SEM for each time point in pmol per minute normalized to the number of cells per well (n = 3).

### Evaluation of the mitochondrial fraction

The effect of sFLG on the mitochondrial fraction was evaluated by confocal microscopy using Mitotracker Green (#M7514; Thermo Fisher). To this end, cells were seeded in 96 well plates and then treated for 7 d with sFLG 5 μg/mL. Cells were loaded for 30 min with Mitotracker (1 µM each), washed in fresh medium and imaged with a Zeiss LSM 880 inverted confocal microscope (63 × objective). Mitotracker was quantified with ImageJ 1.53. Results are presented as the percentage of cell signal *vs.* control or percentage of cell area occupied by mitochondria *vs.* control (> 100 cells). For the internalization study, the same protocol was carried out, with z-stack acquisitions performed on more than 60 slices with a difference of 16.2 μM between slices.

### Determination of Mito Fuel Flex

The XF Mito Fuel Flex Test (#103,260, Seahorse Biosciences) is a method for measuring mitochondrial fuel preferential usage. HaCat cells were incubated for 7 d with sFLG 5 μg/mL in DMEM and then incubated in XFp medium, without glucose, following the same protocol as indicated above. For glutamine and glucose dependency, three baseline OCR measurements were taken for each well in the first 20 min and then BPTES (3 μM), Etomoxir (4 μM) and UK5099 (2 μM) were injected. Seven ECAR values were automatically calculated after each injection by the Seahorse XFp software Wave 2.6. Data are presented as mean ± SEM for each time point in pmol per minute normalized to the number of cells per well (n = 3).

### Ki-67 immunolabeling

Ki-67 positive cells were determined by immunocytochemistry with a specific monoclonal antibody (#sc-15402, SantaCruz BT). Briefly, cells treated for 30 d were seeded in 96 well plates. Medium was removed and cells were fixed for 2 min in cold methanol, blocked and incubated for 60 min with anti-Ki67 antibody (1:500). The cells were then stained with an AlexaFluor-594 anti-mouse (#A-11005, Invitrogen) for 60 min and stained with 1 μg/mL Hoescht (#861405; Sigma-Aldrich). Images were acquired in a Cytation 5 Reader (Biotek) using a 20 × objective and analyzed with ImageJ 1.53.

### Nuclear and cell size study

HaCaT cells were incubated with 5 µg/mL sFLG for 30 d. After treatment, cells were plated in 24-well plates and stained with Hoechst 33,342 Solution (#861,405; Sigma-Aldrich). Bright field and Hoechst images were acquired in a Cytation 5 Reader (Biotek) using a 20 × objective and analyzed with ImageJ 1.53 (> 50 cells/treatment).

### Wound healing assay

Wound healing assays were performed according to the previously reported protocol^23^. Briefly, HaCaT cells were incubated with 5 µg/mL sFLG for 30 d. After treatment, cells were plated in 24-well plates, grown to confluence (24 h) and then serum starved for 48 h. A cross-scratch was made in the cell culture monolayer using a 200 μL pipette tip. Finally, the serum-free medium was removed, the cells were washed with Hanks' solution and fresh medium was added. Images were obtained in a Cytation 5 Reader (Biotek) using a 4 × objective. ImageJ 1.53 was used to calculate the percentage of wound closure by measuring the open area (free of cells) per well immediately after making the scratch and 48 h after (n = 3).

### Statistical analysis

Statistical analysis was conducted using GraphPad Prism 8 (San Diego, CA, USA). Data are presented as mean ± SEM from at least three different experiments. Statistical differences were obtained by Student t-test or one-way ANOVA. Significant differences were considered at: *p < 0.05; **p < 0.01; ***p < 0.001; ****p < 0.0001.

## Supplementary information

Supplementary Information.
