# Supplementary material for: Sublethal exposure of small few-layer graphene promotes metabolic alterations in human skin cells
Source: Sci Rep. 2020 Oct 27;10:18407. doi: 10.1038/s41598-020-75448-0 (PMC7591887; doi:10.1038/s41598-020-75448-0)
Supplement: Supplementary file 1 — Supplementary Information. [file 41598_2020_75448_MOESM1_ESM.docx]

**Supplementary Information. Sublethal exposure of Small Few-Layer Graphene promotes metabolic alterations in human skin cells**

Javier Frontiñan-Rubio^a,b^, M. Victoria Gomez^a^, Viviana Jehová. González^a^, Mario Durán-Prado^b*^ and Ester Vázquez^a,c*^

*^a^ Universidad de Castilla-La Mancha, Instituto Regional de Investigación Científica Aplicada (IRICA), 13071, Ciudad Real, Spain*

*^b^ Universidad de Castilla-La Mancha, Cell Biology area, Department of Medical Sciences, Faculty of Medicine, 13071, Ciudad Real Spain*

*^c^ Universidad de Castilla-La Mancha, Faculty of Chemical Science and Technology, 13071, Ciudad Real, Spain*

* Ester Vázquez ([ester.vazquez@uclm.es](mailto:ester.vazquez@uclm.es)) and Mario Durán-Prado ([mario.duran@uclm.es](mailto:mario.duran@uclm.es))

| **Element** | **Line** | **Conc./**  **mg/l** | **Sigma/**  **mg/l** | **RSD/**  **%** | **LLD/**  **mg/l** | **Net area** | **Backgr.** | **Chi** |
| --- | --- | --- | --- | --- | --- | --- | --- | --- |
| Al | K12 | 1.69 | 0.32 | 19.2 | 0.67 | 578 | 5836 | 1.56 |
| Si | K12 | 66.44 | 0.34 | 0.5 | 0.28 | 52733 | 5676 | 6.27 |
| S | K12 | 8.988 | 0.069 | 0.8 | 0.076 | 26193 | 5509 | 1.75 |
| Cl | K12 | 2.070 | 0.031 | 1.5 | 0.047 | 9929 | 5605 | 3.18 |
| K | K12 | 1.580 | 0.016 | 1.0 | 0.021 | 16560 | 5293 | 1.28 |
| Ca | K12 | 2.494 | 0.017 | 0.7 | 0.017 | 31663 | 5017 | 0.83 |
| Ti | K12 | 10.448 | 0.025 | 0.2 | 0.009 | 253308 | 5814 | 1.49 |
| V (IS) | K12 | 10.000 | 0.022 | 0.2 | 0.010 | 305602 | 10935 | 2.06 |
| Cr | K12 | 0.255 | 0.004 | 1.4 | 0.005 | 9697 | 3813 | 1.29 |
| Mn | K12 | 0.028 | 0.002 | 7.0 | 0.004 | 1304 | 3424 | 1.65 |
| Fe | K12 | 0.294 | 0.003 | 0.9 | 0.003 | 17281 | 2939 | 2.15 |
| Ni | K12 | 0.015 | 0.001 | 6.6 | 0.002 | 1289 | 2929 | 1.03 |
| Cu | K12 | 0.054 | 0.001 | 2.1 | 0.002 | 5441 | 3416 | 1.54 |
| Zn | K12 | 1.457 | 0.004 | 0.3 | 0.001 | 170894 | 3048 | 7.51 |
| Ga | K12 | 0.007 | 0.001 | 9.1 | 0.001 | 983 | 3446 | 1.24 |
| As | K12 | 0.229 | 0.001 | 0.6 | 0.001 | 35048 | 3229 | 1.21 |
| Br | K12 | 0.221 | 0.001 | 0.6 | 0.001 | 41218 | 3422 | 4.22 |
| Rb | K12 | 0.016 | 0.001 | 3.5 | 0.001 | 3260 | 4581 | 18.34 |
| Sr | K12 | Not det. |  |  | 0.001 | 1 | 6628 | 84.91 |
| Pb | L1 | Not det. |  |  | 0.001 | 1 | 2961 | 1.41 |

**Table S. 1.** TXRF of the elements found in sFLG after acid treatment.

**Supplementary Figure 1**

**Supplementary Figure 1**. Elements found in sFLG by TXRF.

**Supplementary Figure 2**

**Supplementary Figure 2**. Individual parameters for basal respiration, ATP production, maximal respiration, spare respiratory capacity, proton leak and non-mitochondrial respiration in cells treated with 5 μg/mL sFLG for 6 h (n = 3).

**Supplementary Figure 3**

**
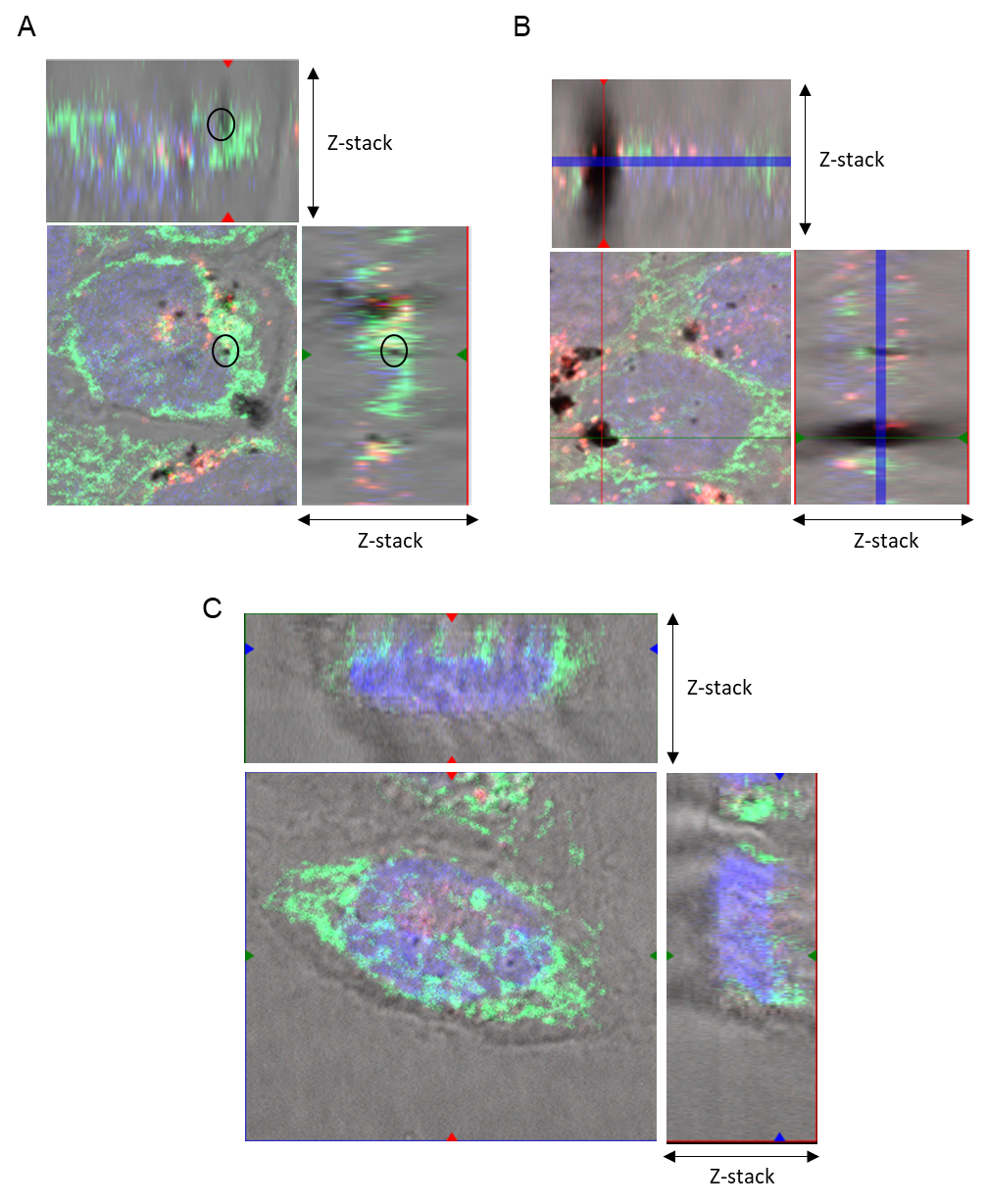
**

**Supplementary Figure 3**. Z-stack of cells stained with MitoTracker Green treated with 5 μg/mL sFLG (black dots) for 24h or control cells. (A) Mitotracker colocalizing with graphene and (B) graphene aggregate deposited on top of a cell. (C) Z-stack of control cells.

**Supplementary Figure 4**


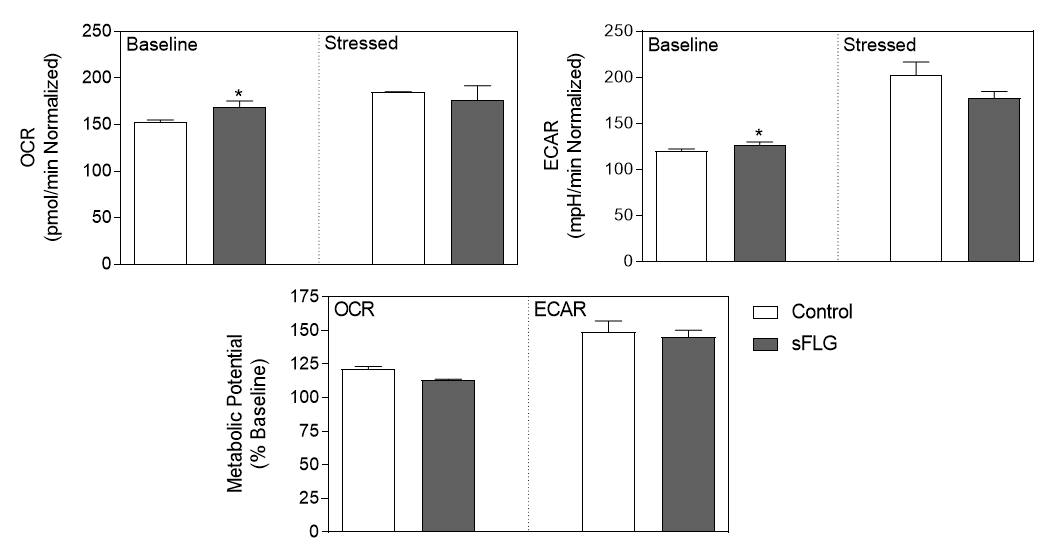


**Supplementary Figure 4**. OCR and ECAR levels under baseline and stressed conditions and metabolic potential in cells treated with 5 μg/mL sFLG for 24 h (n = 4).

**Supplementary Figure 5**


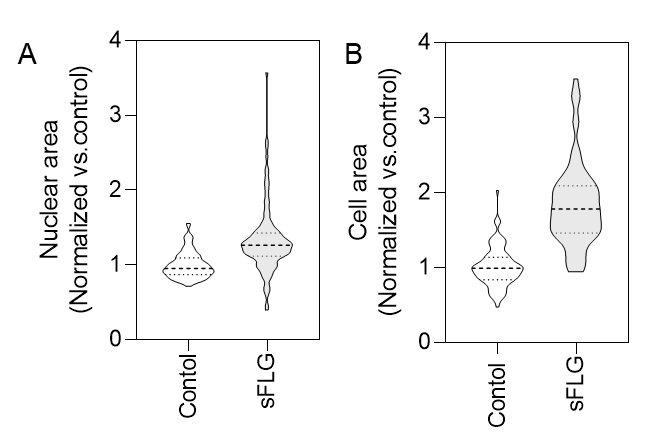


**Supplementary Figure 5**. Nuclear (A) and cell (B) area of cells treated with 5 μg/mL sFLG for 24 h (normalized vs. control) (>50 cells).

**Supplementary Figure 6**


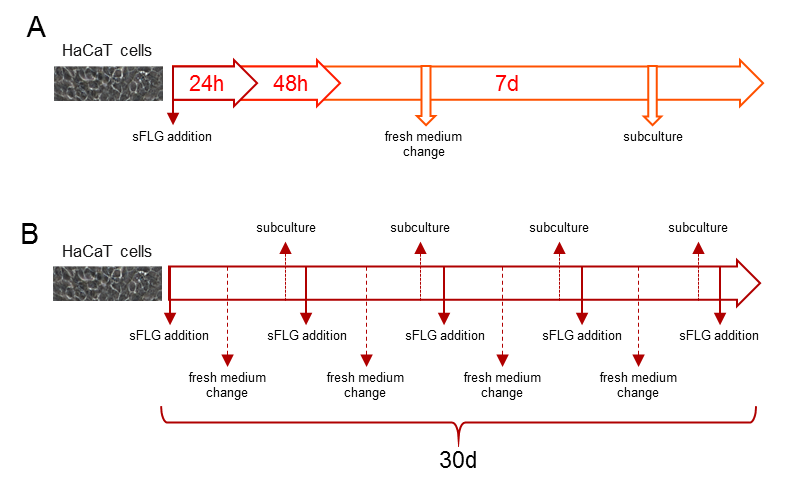


(d1)

(d7)

(d14)

(d21)

(d28)

**Supplementary Figure 6.** Culture of HaCaT cells in sFLG-containing media for 7d (A) and 30d (B). (A) sFLG was added in day one. Fresh medium without sFLG was added on day four. (B) cells were subcultured once a week. sFLG was added on day 1 and medium without sFLG was added on day 4. This sequence was repeated for four weeks.
